# Surgical revision for pancreatojejunostomy stricture: a case series of 14 patients

**DOI:** 10.1186/s12893-022-01767-w

**Published:** 2022-08-19

**Authors:** Feng Guo, Shimeng Huang, Tewodross Getu Wolde, Zipeng Lu, Jianmin Chen, Junli Wu, Wentao Gao, Kuirong Jiang, Yi Miao, Jishu Wei

**Affiliations:** 1 The Pancreas Center of Nanjing Medical University, The First Affiliated Hospital of Nanjing Medical University, Nanjing, China; 2 BenQ Medical Center, The Pancreas Center of Nanjing Medical University, The First Affiliated Hospital of Nanjing Medical University, Nanjing, China

**Keywords:** Pancreatojejunostomy stricture case series, Long-term complications, Pancreatomy, Surgical revision

## Abstract

**Background:**

Pancreatojejunostomy stricture (PJS) is a rare long-term complication of pancreaticojejunal anastomosis. This study aimed to investigate the role of surgery in the management of pancreatojejunostomy strictures.

**Methods:**

The database of the Pancreas Center of Nanjing Medical University was retrospectively screened for patients who underwent a surgical revision for PJS between June 2012 and August 2019, and their clinical characteristics and management modalities were reviewed.

**Results:**

Fourteen consecutive cases were retrieved, the median age at index operation was 41.1 years (19–71). The average time between the two operations was 70.6 months (8–270 months). Index procedures included pancreaticoduodenectomy (PD) (7/14, 50%), pylorus-preserving PD (4/14, 28.6%), Berger procedure (2/14, 14.3%), and middle pancreatectomy (1/14, 7.1%). The diameter of the main pancreatic duct was < 4 mm in all 14 cases, and nine underwent pancreaticojejunostomy (PJ) stenting during the index operation. The most frequent complaints were abdominal pain (6/14, 42.9%), recurrent acute pancreatitis (6/14, 42.9%), pancreatic fistula (1/14, 7.1%), and abdominal distention (1/14, 7.1%). The diagnosis of PJ stricture was confirmed by computed tomography or magnetic resonance imaging in all cases. All patients had a main duct diameter > 5 mm before surgical revision. All patients underwent wedge excision with interrupted one-layer suturing with absorbable sutures and without stent placement. In this series, only one patient required reoperation. Upon follow-up, 11 of 12 patients had complete resolution of the PJ stricture.

**Conclusion:**

PJS is a long-term complication of pancreatojejunostomy. Surgical revision of the anastomosis is a safe and effective treatment modality.

## Introduction

In the last 20 years, pancreatojejunostomy stricture (PJS) has been underreported and not as well studied as pancreatic fistula, hemorrhage, delayed gastric emptying, or even bile leakage. PJS is a rare and long-term complication of pancreatic surgery. Owing to its rarity, most of the available literature is limited to case-reports [1–3]. Recently, surgical techniques and the understanding of pancreatic diseases have tremendously improved. As a result, long-term survival after surgery has progressively improved, and PJS is now more frequently reported [4–6].

While the etiology of PJS remains unknown and uninvestigated, the reported incidence of PJS varies drastically between centers, ranging from 1.4–11.4% to 20–60% [3, 7, 8] and even up to 100% at autopsy [9]. Further studies are needed to validate the true incidence of PJS and establish internationally recognized diagnostic criteria for PJS. Abdominal pain or distention, recurrent acute pancreatitis, and signs of pancreatic endocrine or exocrine insufficiency constitute the major complaints of PJS [5, 10, 11]. Various treatment modalities have been reported for the management of symptomatic PJS, including endoscopic balloon dilatation, robotic surgical revision, percutaneous puncture dilatation, and laser dissection of stricture tissue [2, 12–14]. Here, we analyzed the perioperative, postoperative, and follow-up data from a cohort of patients with PJS managed by open surgical revision at a high-volume pancreatic center.

## Materials and methods

### Patients database

A prospectively maintained pancreatectomy database at the authors’ institution was reviewed and retrospectively analyzed to identify all eligible patients between June 2012 and August 2019. This study was approved by the Institutional Review Board of the First Affiliated Hospital of Nanjing Medical University. Preoperative imaging workup included computed tomography (CT), magnetic resonance imaging (MRI), and/or endoscopic ultrasonography. The following criteria were used to select patients: (1) previous pancreatectomy with pancreaticojejunostomy; (2) clinical symptoms with remnant main duct narrowing at the anastomosis site accompanied by upstream dilation as confirmed by imaging; and (3) recurring abdominal pain, recurrent acute pancreatitis, and unhealed pancreatic fistula. The exclusion criteria were as follows: (1) local recurrence of cancer at the anastomotic site; (2) intraductal papillary mucinous neoplasm (IPMN) recurrence with remnant pancreatic duct dilation; and (3) incomplete medical records or follow-up data.

Patients’ records were reviewed to obtain demographic features, clinical characteristics, imaging results, intraoperative and postoperative data, and pathological findings. Postoperative morbidity, including postoperative pancreatic fistula (POPF), delayed gastric emptying (DGE), and hemorrhage, was assessed according to the International Study Group of Pancreatic Surgery (ISGPS). Postoperative mortality was defined as death before hospital discharge or within 30 days of the operation. Readmission was defined as another admission within 30 days of hospital discharge. This case series has been reported in line with the Process Guideline [15].

### Surgical procedure

**Fig. 1:**
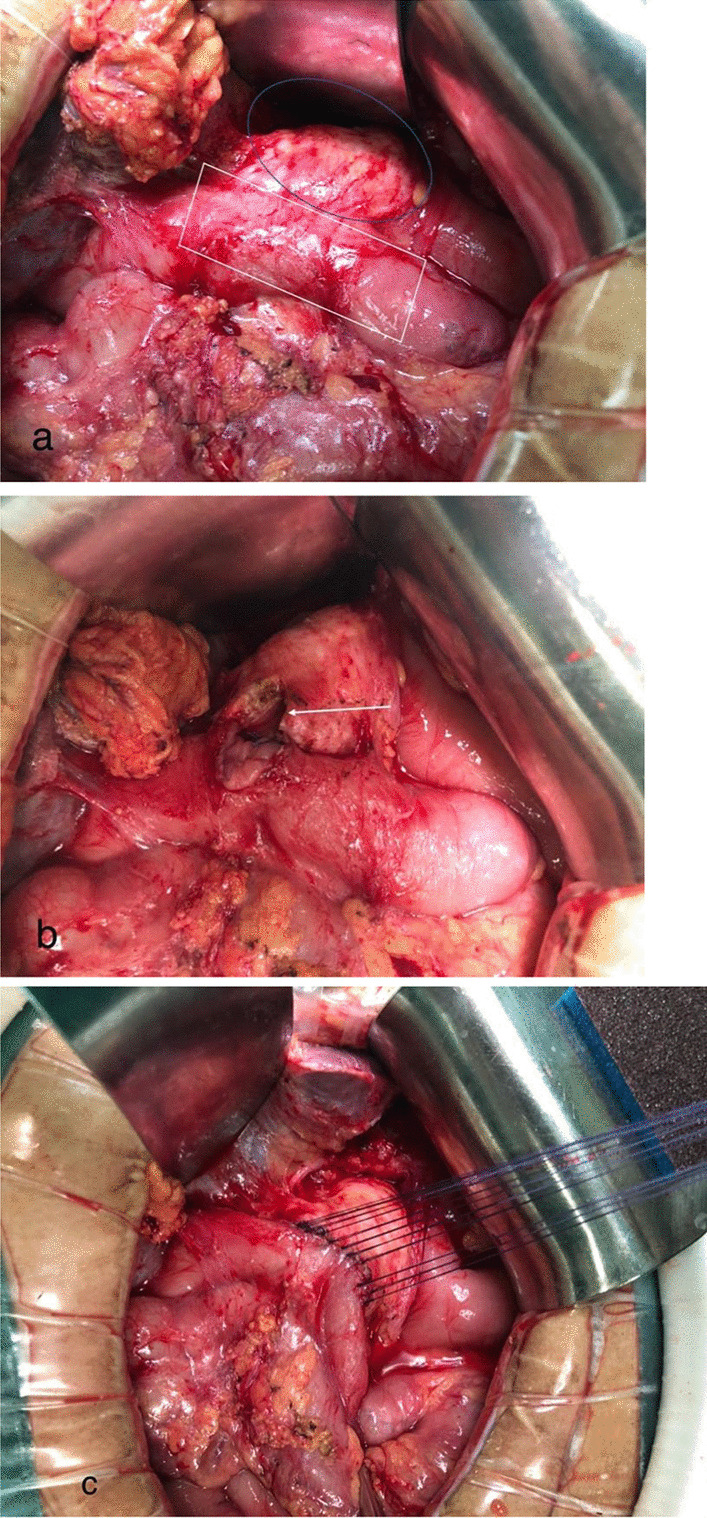
**a** The original pancreaticojejunostomy was dissected, and the posterior wall of the pancreaticojejunostomy did not need to be dissected completely (the blue oval area is the remnant pancreas, the white quadrilateral area is the jejunal input loop). **b** A wedge resection was made to cut out the stricture tissue and expose the dilated remnant main pancreatic duct, then the anterior wall of the main pancreatic duct was cut about 2.0 cm along the longitudinal direction (white arrow). **c** Interrupted suturing with absorbable stitch between the pancreatic parenchyma and the full thickness of the jejunal wall

Surgical revision was warranted in all cases with a dilated residual pancreatic duct along with pancreaticojejunostomy stenosis confirmed by imaging, in which conservative measures failed to alleviate symptoms of epigastric pain or distention and recurrent pancreatitis. In addition, a long-term non-healing pancreatic fistula likely caused by PJ stenosis was another indication for surgery. After a median abdominal incision, the original PJ anastomosis was dissected, while the area, including the superior mesenteric vein/portal vein confluence (SMV/PV) behind the PJ anastomosis, was left untouched. Electrocautery was used to cut the PJ anastomosis from the anterior to the posterior wall. It is worth noting that the posterior wall of the pancreaticojejunostomy should not be dissected because the portal vein often lies behind it (Fig. 1a). Afterward, wedge resection was performed to cut out the stricture tissue and expose the dilated remnant main pancreatic duct (MPD). A 2.0 cm opening was created on the anterior wall of the remnant MPD depending on the direction of the main duct, and a corresponding incision was made on the anterior wall of the jejunal limb (Fig. 1b). The newly made PJ section was sutured in an interrupted fashion using absorbable sutures between the pancreatic parenchyma and the full thickness of the jejunal wall (Fig. 1c).

Finally, a closed suction drain was placed on the anterior wall of the new PJ.

### Postoperative complications

The postoperative assessment included the occurrence of pancreatic fistulae (PF), abdominal infection, hemorrhage, DGE, pancreatitis, wound infections, and mortality. PF was defined and graded according to the updated ISGPS definition (2016) [16]. Abdominal infection was confirmed when the abdominal drainage fluid cultures were positive.

### Follow-up

Follow-up was carried out by reviewing hospital and office medical records and direct telephone contact biannually by full-time follow-up staff.

### Statistical analysis

Continuous data were expressed as mean ± SD or median (range) and categorical data were presented as number (percent %). All statistical analyses were performed using Stata/SE 10.0 for Windows.

## Results

### Patient and preoperative characteristics

Fourteen patients were enrolled in this study. Patients’ demographic data and index operation data are shown in Table 1.

**Table 1:** Patient demographics index operation data and pathology results of

Patient No.	Age	Sex	PFPG	DM	Steatorrhea	Index surgical approach	Anastomotic method of index operation	Stent	Pathology results of index operation	Pathology results of
1	38	M	4.43	N	N	MP	End-to-side, two-layer	N	SCN	Chronic inflammation
2	64	M	4.58	N	N	Beger	End-to-side, two-layer	Y	MCN	Chronic inflammation
3	38	M	4.47	N	N	PD	End-to-side, two-layer	N	MCN	Chronic inflammation
4	74	F	8.44	Y	N	PD	End-to-side, two-layer	N	Duodenal papilla a denocarcinoma	Chronic inflammation
5	50	M	6.16	N	N	PD	End-to-side, two-layer	Y	Pancreatic neuroendocrine tumour	Neuroendocrine tumor
6	34	F	4.94	N	N	PPPD	End-to-side, one-layer	Y	IOPN	Chronic inflammation
7	48	M	6.28	N	N	PD	End-to-side, two-layer	Y	Duodenal neuroendocrine tumor	Chronic inflammation
8	58	M	4.47	N	N	PD	End-to-side, two-layer	N	IPMN with focal canceration	Traumatic neurofibroma
9	37	F	5.09	N	N	PD	End-to-side, two-layer	Y	Duodenal GIST	Chronic inflammation
10	71	M	4.98	N	N	PD	End-to-side, unknown	Y	Chronic pancreatitis	Chronic inflammation
11	40	M	5.47	N	N	Beger	End-to-side, two-layer	N	Chronic pancreatitis	Chronic inflammation
12	56	M	4.79	N	N	PPPD	End-to-side, unknown	Y	Adenoma of duodenal papilla with HGIEN	Chronic inflammation
13	23	F	4.83	N	Y	PPPD	End-to-side, one-layer	Y	SPT	Chronic inflammation
14	33	F	4.12	N	Y	PPPD	End-to-side, one-layer	Y	SPT	Chronic inflammation

*M* male, *F* female, *PFPG* preoperative fasting plasma glucose, *DM* diabetes mellitus, *N* no, *Y* yes, *MP* middle pancreatectomy, *PD* pancreaticoduodenectomy, *PPPD* pylorus-preserving pancreaticoduodenectomy, *SCN* serous cystic neoplasm, *MCN* mucinous cystic neoplasm, *IOPN* intraductal oncocytic papillary neoplasm, *IPMN* intraductal papillary mucinous neoplasm, *GIST* gastrointestinal stromal tumor, *HGIEN* high-grade intraepithelial neoplasia, *SPT* solid pseudopapillary tumor

Whipple, Beger, and central pancreatectomy accounted for 78.6% (11/14), 14.3% (2/14), and 7.1% (1/14), respectively. All cases in this study received an end-to-side PJ, and all had an MPD diameter of less than 4 mm. The two-layer duct-to-mucosa technique was used in nine patients, while three patients had a one-layer PJ anastomosis. The method of anastomosis was not accurately recorded in two patients. At the time of the index operation, non-absorbable plastic pancreatic duct internal stents were placed in nine of the 14 cases. Even so, none of the stents could be found during the revision surgery. Histopathological analysis of tumor specimens from the index operation indicated benign (11/14, 78.6%), low-grade malignant (2/14, 14.3%), and malignant tumors (1/14, 7.1%). Detailed information is shown in Table 1.

**Fig. 2:**
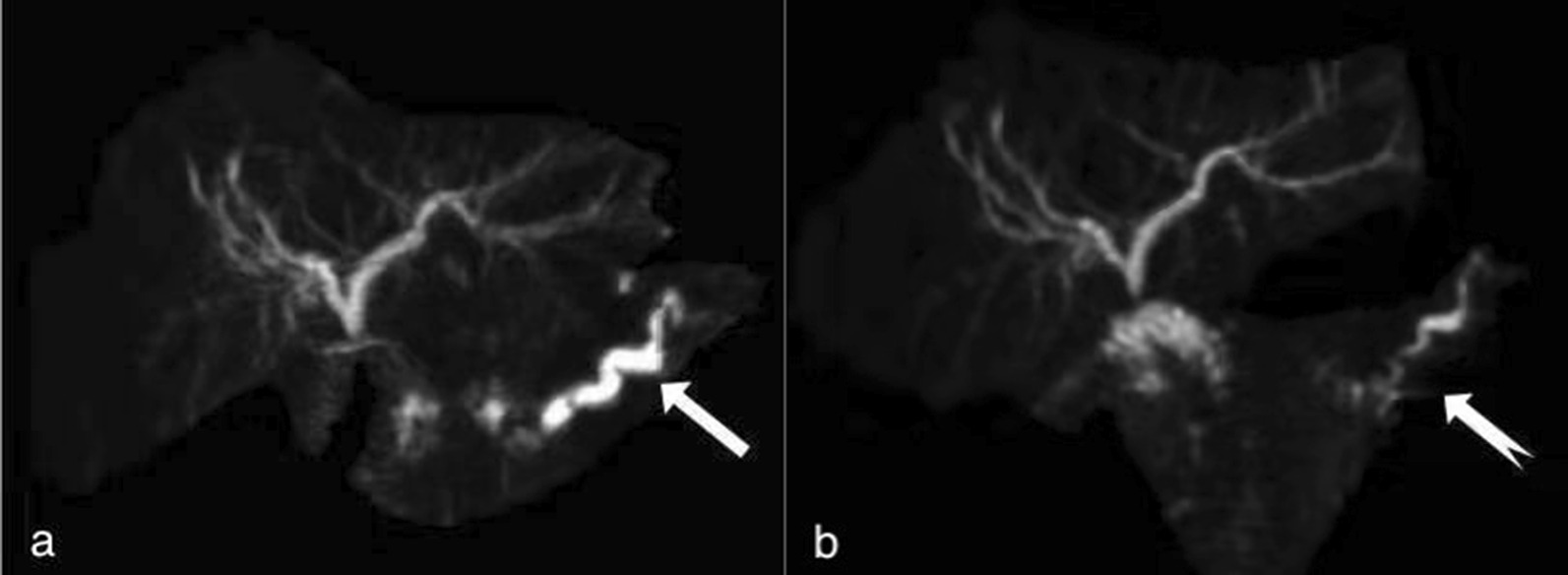
**a** The main duct of remnant pancreas was dilated obviously (white arrow) before operative revision. **b** 1 week after the surgical revision, the dilation of the remnant pancreatic duct relieved significantly (dovetail arrow))

The average time from presentation of the earliest clinical symptoms to surgical revision was 54.4 months. The average time between the two operations was 70.6 months (8–270 months). The most frequent complaints were abdominal pain (6/14, 42.9%), recurrent acute pancreatitis (6/14, 42.9%), pancreatic fistula (1/14, 7.1%), and abdominal distention (1/14, 7.1%). Almost all patients underwent CT (13/14, 92.6%) or MRI (8/14, 57.1%) at least once (Fig. 2). Six patients underwent endoscopic retrograde cholangiopancreatography (ERCP), and three patients required reintervention. A PJ stent had to be placed more than twice in two cases, and one case had an unsuccessful stent placement.

### Operative procedures and outcomes

The mean diameter of the MPD assessed by preoperative imaging was 5.1 ± 3.0 mm. One case of tumor recurrence was found at the PJ site, with no evidence of IPMN in the residual pancreas. Detailed intraoperative and postoperative data related to PJ revision are shown in Table 2.

**Table 2:** Details of the revision surgery

	Mean ± SD
Diameter of the main pancreatic duct (mm)	5.1 ± 3.0
Operating time (min)	132 ± 69
Blood loss (mL)	114 ± 49
Mortality	0/0%
Major complications	2/14.3%
Pancreatic fistula (grade B)	1/7.1%
Intra-abdominal hemorrhage	1/7.1%
Reoperation	1/7.1%
Postoperative hospital length of stay (days)	15 ± 9
Hospitalization costs	¥58,432 ± 24,437 ($8253 ± 3451)

In nearly all cases, histopathological reports of the resected stricture tissue indicated chronic inflammation. In addition, traumatic neuroma and local recurrence of neuroendocrine tumors were also detected (Table 1).

### Follow-up data

Follow-up data were finalized and updated in January 2020, with complete information acquired in 12 of the 14 cases. The average follow-up time was 59.1 months, with a median of 43.0 months. In these 12 cases, five patients had complete ceasing of recurrent acute pancreatitis; six of the seven patients with abdominal pain were symptom-free, while one patient only had partial resolution of pain.

## Discussion

PJS is a rare long-term complication of pancreaticojejunostomy and is seldom observed or reported. The first case of PJS was published in 1966, two decades after the first Whipple operation was performed [17]. Thus far, sufficiently large reports on PJS are lacking. Until 2017, only 18 studies qualified for meta-analysis, with the largest one consisting of only 27 patients [3]. Unlike choledochojejunostomy, for which diagnosis and management are relatively well established, PJS has not benefited from the same widespread attention. Owing to the fact that this complication is barely recognized by surgeons and due to the relatively short survival period of pancreatic cancer patients after surgery, late complications, such as complete or partial PJS, remain vastly overlooked and have a limited time to surface.

The exact etiology of PJS remains uncertain, with contradictory hypotheses reported in the literature [18–20]. Possible etiological factors, such as pancreatic stump texture, ischemia, or anastomotic suturing technique, have yet to be assessed. In the majority of reports, PJS was predominant in patients with low-grade malignancy or benign tumors. Thus, the main duct was not dilated during index operation [4, 21, 22]. One possible explanation is that patients with low-grade malignancies or benign diseases have long postoperative survival.

Currently, there is no consensus or guideline for the diagnosis and treatment of PJS. Diagnosis is mainly based on the clinical manifestations and imaging findings described in series [10, 23, 24]. The main clinical manifestations of PJS include abdominal pain, distention, and recurrent pancreatitis, which is consistent with the results of this study [25, 26]. Unfortunately, important diagnostic criteria, such as accurate measurement of MPD dilation and assessment of exocrine function of the pancreas, were not taken into consideration in various published reports. Several management modalities have been described for the treatment of PJS, including percutaneous puncture-guided CT, endoscopy, surgery, and laser dissection [2, 4, 12, 27]. Nevertheless, there is no broad consensus regarding the therapeutic strategies for PJS. Notably, several important issues still exist and need to be addressed. For example, questions concerning the benefit of early intervention in managing asymptomatic PJS with or without exocrine insufficiency. Moreover, no clear indications exist for pharmacological treatment, endoscopic intervention, or surgical revision of PJS. With the advancement of pancreatic surgical techniques, improvement of neoadjuvant chemotherapy, and targeted therapy, an increasing number of benign or low-grade malignant tumors are detected earlier and operated on with longer survival periods after surgery. Therefore, it must be assumed that the occurrence of PJS will only increase in the foreseeable future. An international consensus for the diagnosis and management of PJS should be established in the near future.

Despite limited published data, endoscopy and surgery constitute the two major treatment modalities for PJS. Due to reconstruction of the digestive tract after pancreatic surgery, the afferent limb measures between 30 and 50 cm, which renders insertion of a conventional endoscope into the PJ tremendously difficult. The success rate of endoscopic management of PJS varies dramatically among reports. The success of e-ERP has been reported to be as low as 8%, even when performed by experienced endoscopists [28] Nonetheless, Kikuyama et al. achieved a 100% success rate [20]. In the majority of reports, the failure rate of ERCP treatment for PJS fluctuates between 75 and 80% [3, 20, 29] Double balloon endoscopy (DBE) has been shown to facilitate and improve the success rate of ERCP after pancreaticoduodenectomy. Unfortunately, the failure rate of endoscopic treatment of PJS remains high owing to several factors. Some common reasons for failure include difficulty in reaching the PJ loop, inability to identify the PJ, and failed cannulation of the MPD due to severe stenosis [30–32]. In recent reports, the rendezvous technique has had a high success rate for stenting the stricture of PJ [20]. Presumably, this technique should be superior to traditional ERCP and DBE techniques. Furthermore, the complication rate, procedure cost, and long-term results of endoscopic procedures need to be compared to surgery in this setting [4].

Compared with endoscopic treatment, surgical revision is safe and effective. In the PJS, the main duct is dilated, and the parenchyma is hardened by long-term duct obstruction. Evidently, these two factors decrease the technical difficulty, making surgical intervention simple and safe. Likewise, in many reports, surgical redo-PJ appears to be safe and feasible by professional pancreatic surgeons. Reported intraoperative blood loss was less than 200 mL, and none of the patients suffered from POPF after surgery [5, 11] In line with previous reports, the operation time, blood loss, and postoperative complications in our study confirmed that redo-PJ is a relatively straightforward and safe procedure when performed by professional pancreatic surgeons [4, 13, 22, 27]. More importantly, surgical revision has a better long-term outcome when compared to endoscopic treatment. In a report by Stephania et al., both patients who underwent redo-PJ were asymptomatic for more than 4 years after surgery [25]. Similarly, Cioffi et al. reported that 78% of patients who underwent PJ revision surgery experienced a complete resolution of symptoms during a median follow-up of 30 months [4]. Another study described excellent pain relief in 5/6 patients and average pain relief in one patient during a median follow-up of 36 months (16–84 months) [11]. Likewise, in our study, 92% (11/12) of patients had complete remission of clinical symptoms at a median follow-up time of 43 months. Unlike surgical revision, the available literature on endoscopic treatment is mostly limited to case reports with a shorter follow-up time (6 − 8 months) [23, 24, 33]. Compared to surgery, lower pain relief was reported at the 24-month follow-up, with relief in only 2/3 of patients [19].

The current study is among the few reports that focus on the surgical management of PJS. However, it has some notable drawbacks owing to the retrospective nature of the study, with inherent limitations in its design. First, the analysis of a surgical revision group without comparison to an endoscopic treatment group as a control cohort makes the evidence less significant. Moreover, all cases were from a specialized tertiary pancreatic center, limiting its applicability in the general surgery department.

In conclusion, endoscopic treatment may have a future role for patients with symptomatic PJS, and the existing evidence favors surgical revision and promotes surgical revision in centers of expertise in pancreatic surgery as the recommended management for PJS at present.

## Data Availability

All data generated or analysed during this study are included in this published article.
